# Plantar ulcer as an atypical manifestation of cutaneous leishmaniasis^☆^^☆☆^

**DOI:** 10.1016/j.abd.2020.06.015

**Published:** 2021-03-15

**Authors:** Fernanda de Carvalho Tironi, Gustavo Uzeda Machado, Sergio Marcos Arruda, Paulo Roberto Lima Machado

**Affiliations:** a Universidade Federal de São Paulo, São Paulo, SP, Brazil; b Escola Bahiana de Medicina, Salvador, BA, Brazil; c Fundação Oswaldo Cruz, Salvador, BA, Brazil; d Universidade Federal da Bahia, Salvador, BA, Brazil

**Keywords:** Cutaneous leishmaniasis, Leishmaniasis, Plantar ulcer, Ulcer

## Abstract

Cutaneous leishmaniasis is characterized by ulcers with raised edges and a granular bottom, mainly on the lower limbs. This is a case report of a male patient with an ulcer on the left plantar region. The diagnosis was confirmed by positive PCR for *L. braziliensis* and the presence of amastigotes of *Leishmania sp*. in the histopathological examination. After treatment with Glucantime, the patient showed full healing of the ulcer. The unusual location of the ulceration calls attention to atypical presentations of leishmaniasis, and the importance of histopathological examination and PCR, leading to the appropriate diagnosis and treatment.

## Introduction

American Tegumentary Leishmaniasis (ATL) is an endemic, neglected, and important disease in Brazil. ATL, whose main agent in our country is *Leishmania braziliensis*, it is characterized by several clinical manifestations, resulting in mucocutaneous involvement, in addition to disseminated and atypical forms.^1^, ^2^ The disease pathogenesis is the result of the interaction between the genetic polymorphism of the parasite, host immune response, and environmental conditions, resulting in different clinical presentations.^2^, ^3^

Cutaneous Leishmaniasis (CL) is the most prevalent clinical presentation and is characterized by one or more rounded ulcers, with a granular bottom and raised edges, located mainly in exposed parts of the skin, corresponding to the parasite inoculation site by the vector insect.^1^, ^2^, ^3^ The presence of lesions in less accessible body areas such as the scalp, genital region, or palmoplantar location, constitutes an atypical manifestation of the disease.^2^ Plantar ulcers in the absence of disseminated disease in ATL are extremely uncommon and should be differentiated from other etiologies, including leprosy and perforating disease associated with diabetes, thus being an important differential diagnosis in endemic areas of leishmaniasis.^4^, ^5^

## Case report

**Figure 1:**
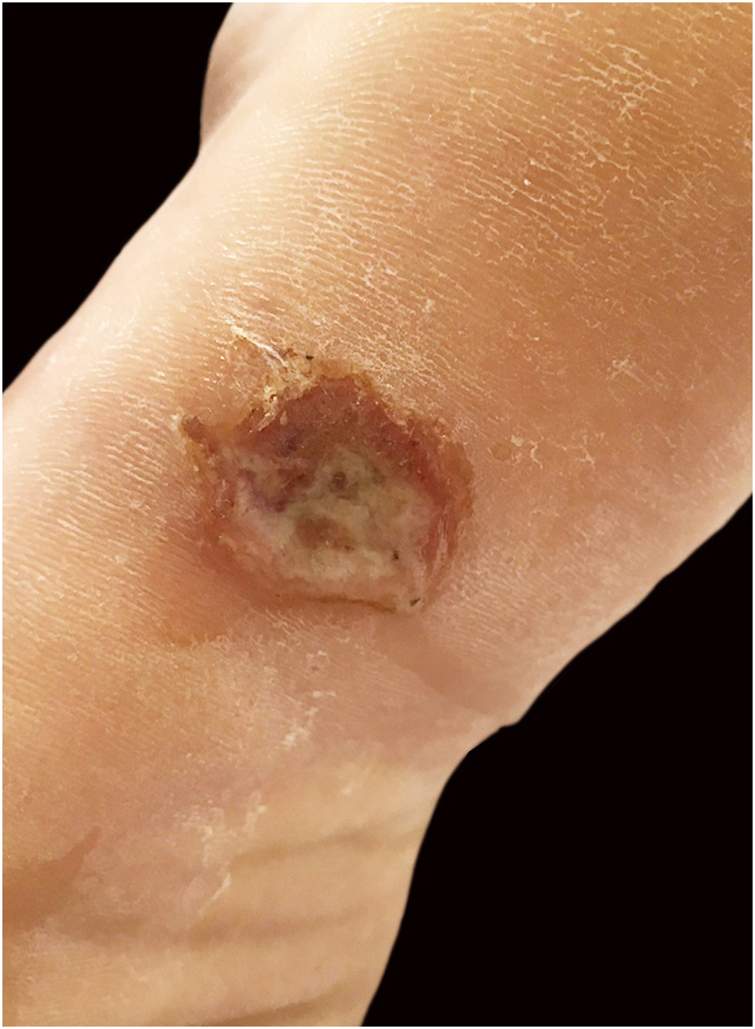
Ulcer with raised edges on the left plantar region.

**Figure 2:**
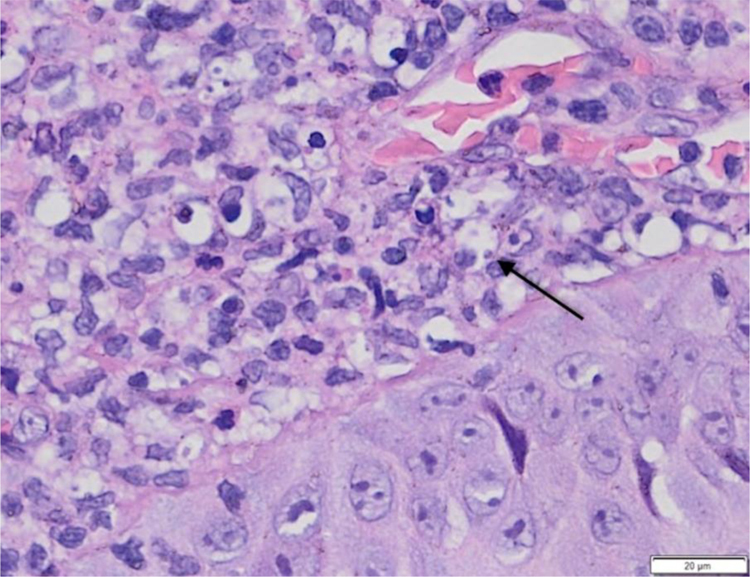
Anatomopathological examination of a plantar ulcer showing dermal infiltrate and amastigotes of *L. braziliensis* inside macrophages (arrow). (Hematoxylin & eosin, ×100).

A twenty-one-year-old male patient was referred to the Corte de Pedra Leishmaniasis Reference Center in the municipality of Presidente Tancredo Neves, in the state of Bahia, Brazil. He was previously healthy and had no report of chronic diseases or previous history of ulcerated skin lesions suspected of leishmaniasis. He observed the sudden appearance of a papular and ulcerated lesion in the axillary region with approximately 35 days of evolution. Concomitantly, he had an ulcerated lesion with raised edges on the left plantar region, associated with fever and myalgia. He subsequently evolved with the formation of two erosive lesions in the groin and left buttock regions 20 days after the onset of the manifestations, with lymphadenopathy in the left inguinal region. The physical examination showed a rounded ulcer with a granular bottom on the plantar region of the left foot measuring 11 × 15 mm (Fig. 1) and three ulcerated papules in the left axilla, buttock, and groin region. The biopsy of the plantar ulcer edge showed positive PCR for *L. braziliensis* and the histopathological analysis showed the presence of amastigotes of *L. braziliensis* (Fig. 2). Treatment with meglumine antimoniate was implemented for 30 days, and the patient showed complete regression of the skin lesions and full healing of the plantar ulcer.

## Discussion

The classical presentation of CL comprises a round ulcer with raised edges and a granular bottom, located mainly in exposed regions, which correspond to the parasite inoculation site by the vector insect. Ulceration on the plantar region is characterized as an atypical manifestation of cutaneous leishmaniasis. Few reports have been described in the literature since it represents an area of the skin where the thickness of the corneal layer and difficulty of access make inoculation by the vector unfeasible.

During the investigation of the condition, it is important to consider differential diagnoses such as leprosy, vasculitis, and chronic ulcers due to vascular insufficiency or diabetes. The histopathological analysis with evidence of the protozoan presence in the affected tissue and confirmatory PCR analysis are essential in these cases for diagnostic confirmation and implementation of adequate curative treatment.

## Financial support

None declared.

## Authors’ contributions

Fernanda de Carvalho Tironi: Study design and planning; drafting and editing of the manuscript; critical review of the manuscript.

Gustavo Uzeda Machado: Approval of the final version of the manuscript; effective participation in the research orientation.

Sergio Marcos Arruda: Collection, analysis, and interpretation of data; intellectual participation in the propaedeutic and/or therapeutic conduct of the studied cases.

Paulo Roberto Lima Machado: Approval of the final version of the manuscript; collection, analysis, and interpretation of data; effective participation in the research orientation; intellectual participation in the propaedeutic and/or therapeutic conduct of the studied cases; critical review of the manuscript.

## Conflicts of interest

None declared.
